# Foot pain: common, of consequence, and consulted about

**DOI:** 10.1186/1757-1146-4-S1-A7

**Published:** 2011-05-20

**Authors:** Edward Roddy

**Affiliations:** 1 Arthritis Research UK Primary Care Centre, Keele University, Staffordshire, ST5 5BG, UK

## 

Foot pain is highly prevalent in the community. Foot pain and ankle pain occurring on most days are reported by 25% and 15% of older adults, respectively. Both foot and ankle pain are more prevalent in women than men but no consistent relationship with age has been identified. Pain within the foot most commonly occurs at the toes or forefoot, followed by the arch or ball, with the heel and hindfoot least frequently affected.

Foot pain is commonly associated with locomotor disability. Almost two-thirds of adults aged 50 years and over with foot pain report problems related to physical function. Eight percent of older adults without disabling foot pain experience its onset over a three-year period whereas resolution appears to be uncommon, with 70% of those with disabling symptoms still having them three years later. Onset of physical impairment associated with foot pain most commonly relates to increased reliance on vehicular transport, and difficulty walking and standing. Furthermore, foot pain is also associated with poor balance and risk of falling.

Although musculoskeletal foot problems account for a substantial number of consultations in primary care, it appears that only a minority of people with foot problems consult about them. Eight percent of primary care musculoskeletal consultations (290 primary care consultations per 10,000 registered patients per year) involve musculoskeletal foot problems. However, only 20% of people with musculoskeletal foot problems see their family doctor over a three-year period and a similar proportion of those with foot pain see a podiatrist over twelve months. Primary care consultation with a musculoskeletal foot problem is more likely in those with foot pain, those who consult frequently about other problems, and those who believe effective treatments are available. Most primary care consultations for musculoskeletal foot problems are categorized by family doctors using non-specific terms describing pain location, eg foot pain or ankle pain, with specific diagnostic codes, eg plantar fasciitis, used less frequently.

In summary, foot pain is a common problem both in the community and in primary care, and frequently associates with locomotor disability. However, only a small proportion of those with musculoskeletal foot problems consult about them. Further studies are needed to explore why people choose to consult a particular health care professional about their foot problem, how the decision to consult can be influenced, and how foot pain and problems are managed in different healthcare settings.

